# Diversity of tsetse flies and trypanosome species circulating in the area of Lake Iro in southeastern Chad

**DOI:** 10.1186/s13071-021-04782-7

**Published:** 2021-06-02

**Authors:** Djoukzoumka Signaboubo, Vincent Khan Payne, Ibrahim Mahamat Alhadj Moussa, Hassane Mahamat Hassane, Petra Berger, Soerge Kelm, Gustave Simo

**Affiliations:** 1 Molecular Parasitology and Applied Entomology Unit, Department of Biochemistry, Faculty of Science, University of Dschang, PO Box 67, Dschang, Cameroon; 2 Centre for Biomolecular Interaction Bremen, Department of Biology and Chemistry, University of Bremen, Bremen, Germany; 3 Laboratory of Biology and Ecology (LABEA), Department of Animal Biology, Faculty of Science, University of Dschang, PO Box 067, Dschang, Cameroon; 4 Institut de Recherche en Elevage Pour Le Développement, BP 433, Rue Farcha, N’Djamena, Chad

**Keywords:** Trypanosome infections, Tsetse fly, AAT, Lake Iro, Chad

## Abstract

**Background:**

African trypanosomiases are vector-borne diseases that affect humans and livestock in sub-Saharan Africa. Although data have been collected on tsetse fauna as well as trypanosome infections in tsetse flies and mammals in foci of sleeping sickness in Chad, the situation of tsetse fly-transmitted trypanosomes remains unknown in several tsetse-infested areas of Chad. This study was designed to fill this epidemiological knowledge gap by determining the tsetse fauna as well as the trypanosomes infecting tsetse flies in the area of Lake Iro in southeastern Chad.

**Methods:**

Tsetse flies were trapped along the Salamat River using biconical traps. The proboscis and tsetse body were removed from each fly. DNA was extracted from the proboscis using proteinase K and phosphate buffer and from the tsetse body using Chelex 5%. Tsetse flies were identified by amplifying and sequencing the cytochrome* c* oxydase I gene of each tsetse fly. Trypanosome species were detected by amplifying and sequencing the internal transcribed spacer 1 of infecting trypanosomes.

**Results:**

A total of 617 tsetse flies were trapped; the apparent density of flies per trap per day was 2. 6. Of the trapped flies, 359 were randomly selected for the molecular identification and for the detection of infecting trypanosomes. *Glossina morsitans submorsitans* (96.1%) was the dominant tsetse fly species followed by *G. fuscipes fuscipes* (3.1%) and *G. tachinoides* (0.8%). Four trypanosome species, including *Trypanosoma vivax*,* T. simiae*, *T. godfreyi* and *T. congolense* savannah, were detected. Both single infection (56.7%) and mixed infections of trypanosomes (4.6%) were detected in *G. m. submorsitans*. The single infection included *T. simiae* (20.5%), *T. congolense* savannah (16.43%), *T. vivax* (11.7%) and *T. godfreyi* (9.8%). The trypanosome infection rate was 61.4% in *G. m. submorsitans*, 72.7% in *G. f. fuscipes* and 66.6% in *G. tachinoides*. Trypanosome infections were more prevalent in tsetse bodies (40.6%) than in the proboscis (16.3%).

**Conclusion:**

This study revealed the presence of different tsetse species and a diversity of trypanosomes pathogenic to livestock in the area of Lake Iro. The results highlight the risks and constraints that animal African trypanosomiasis pose to livestock breeding and the importance of assessing trypanosome infections in livestock in this area.
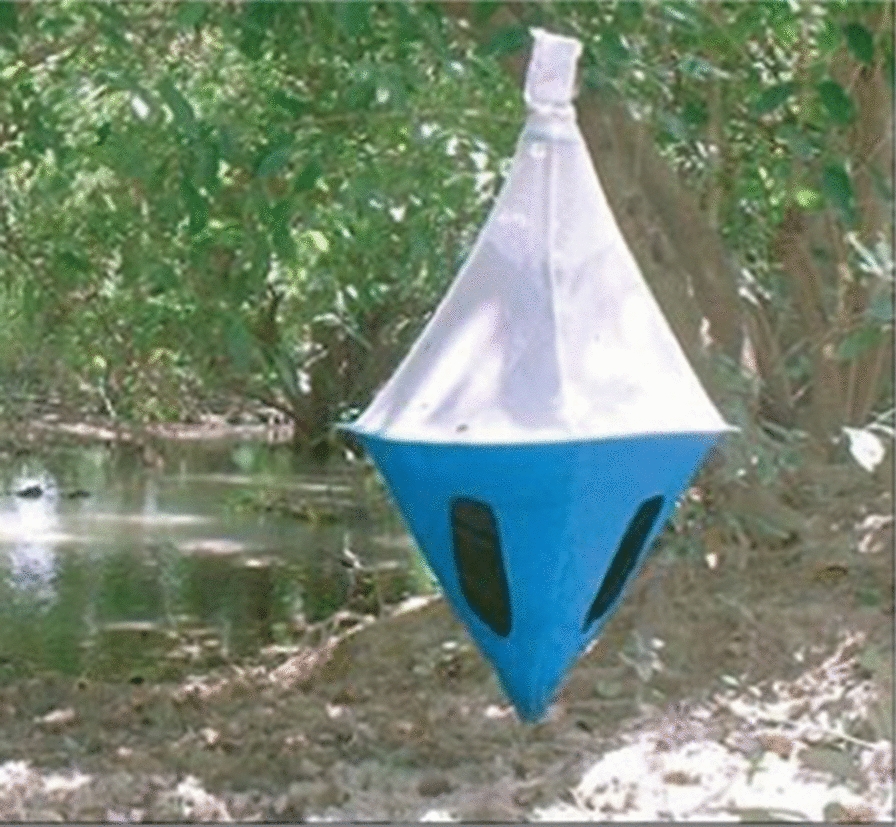

## Background

African trypanosomiases are parasitic diseases that are widely spread throughout sub-Saharan Africa. They are caused by African trypanosomes and induce morbidity and mortality both in humans and animals. Human African trypanosomiasis (HAT), or sleeping sickness, is a neglected tropical disease (NTD) caused by two trypanosome subspecies: *Trypanosoma brucei gambiense*, which is responsible for the chronic form of the disease in western and central African regions, and *T. b. rhodesiense*, which induces the acute form of the disease in southern and eastern African regions [1].

Animal African trypanosomiasis (AAT), or nagana, is caused by several trypanosome species or subspecies, including *T. vivax*,* T. simiae*,* T. b. brucei* and *T. congolense.* Amongst these animal trypanosomes, *T. congolense* is considered to be the most important pathogen due to its economic impact on animal production [2]. In about 38 sub-Saharan African countries with a high potential of producing meat, milk and food crops, AAT is recognized as an important constraint to agriculture, livestock production, food security and animal health. Globally, about 55 million heads of cattle, 30 million sheep and 40 million goats are at risk of trypanosome infections [3]. These infections reduce milk production by 10–40%, the number of cattle by 10–50% and agricultural production by up to 2–10%. Moreover, direct and indirect losses resulting from the impact of AAT on the African economy have been estimated to be 5 billion euro annually [4]. Controlling AAT will enable the agricultural industry to benefit by about US$1300 million per annum [5]. The Pan African Tsetse and Trypanosomiases Eradication Campaign (PATTEC) was launched about 20 years ago with the aim to overcome the economic losses deriving from trypanosome infections in livestock [6]. Achieving the PATTEC goal requires updating knowledge on tsetse distribution and trypanosome infections in both tsetse flies and mammals in different ecological settings. Knowledge of these factors is a pre-requirement for designing and implementing effective control operations against AAT since it could help to identify and map zones presenting high transmission risks where control operations must be deployed [7].

In tsetse-infested areas, transmission of trypanosomes relies mainly on tsetse flies, which are cyclic vectors of African trypanosomes. Given the role played by tsetse flies (*Glossina* spp.) in the life-cycle of African trypanosomes, it is obvious that data on trypanosome infections in tsetse flies could contribute to an understanding of the transmission of these parasites. In this context, considerable data have been generated on both tsetse fauna and trypanosome infections in tsetse and mammals in foci of sleeping sickness and in most tsetse-infested areas that are subjected to intensive animal breeding [8–10]. However, updating data on tsetse fauna and trypanosome species infecting tsetse and mammals remains important for understanding the current epidemiological situation of tsetse transmitted-trypanosomes in some tsetse-infested areas, such as the National Parks, the lakes and their surrounding areas. Although most of these areas provide bioclimatic conditions favourable for tsetse development and trypanosome transmission, the tsetse fauna as well as the trypanosome infections in tsetse, livestock and wild animals remain not well understood. Identifying the *Glossina* fauna as well as trypanosome infections in tsetse and mammals appears fundamental for the understanding of the epidemiology of AAT in different ecological settings and for the achievement of the PATTEC goal of eliminating tsetse-transmitted trypanosomes in Africa.

The southern part of Chad is part of the tsetse-infested area of the Soudano-Sahelian region. Although livestock breeding can be considered to be one of the most important economic activities, with more than 93.8 million heads of cattle, sheep, goats, camels, donkey, horses and pigs being bred by inhabitants of tsetse-infested areas [11], few investigations have been carried out on parasitic diseases, such as AAT, that affect livestock health and animal production. Entomological investigations in sleeping sickness foci and some tsetse-infested areas in the same trapping sites have reported the presence of the tsetse fly species *Glossina tachinoides*,* G. fuscipes fuscipes* and *G. morsitans submorsitans* [12, 13]. Although a variety of tsetse species with trypanosome infections have been reported in sleeping sickness foci and in some tsetse-infested areas of Chad, the distribution of infecting trypanosome species or subspecies is still not well understood. Filling knowledge gaps on tsetse-transmitted trypanosomes requires investigations aimed at updating data on *Glossina* fauna as well as trypanosome infections in various tsetse-infested areas, such as the Lake Iro region that receives, yearly during the dry season, many transhumant herders and nomads searching for grazing and water for their livestock. Although this area provides suitable habitats for tsetse development and trypanosome transmission, the identity of tsetse fauna and trypanosomes circulating in the area of Lake Iro remain largely unknown.

This study was designed to generate entomological and parasitological data on tsetse fauna and trypanosome infections in tsetse flies caught in tsetse infested areas of Lake Iro in the south of Chad.

## Methods

### Study area

**Fig. 1:**
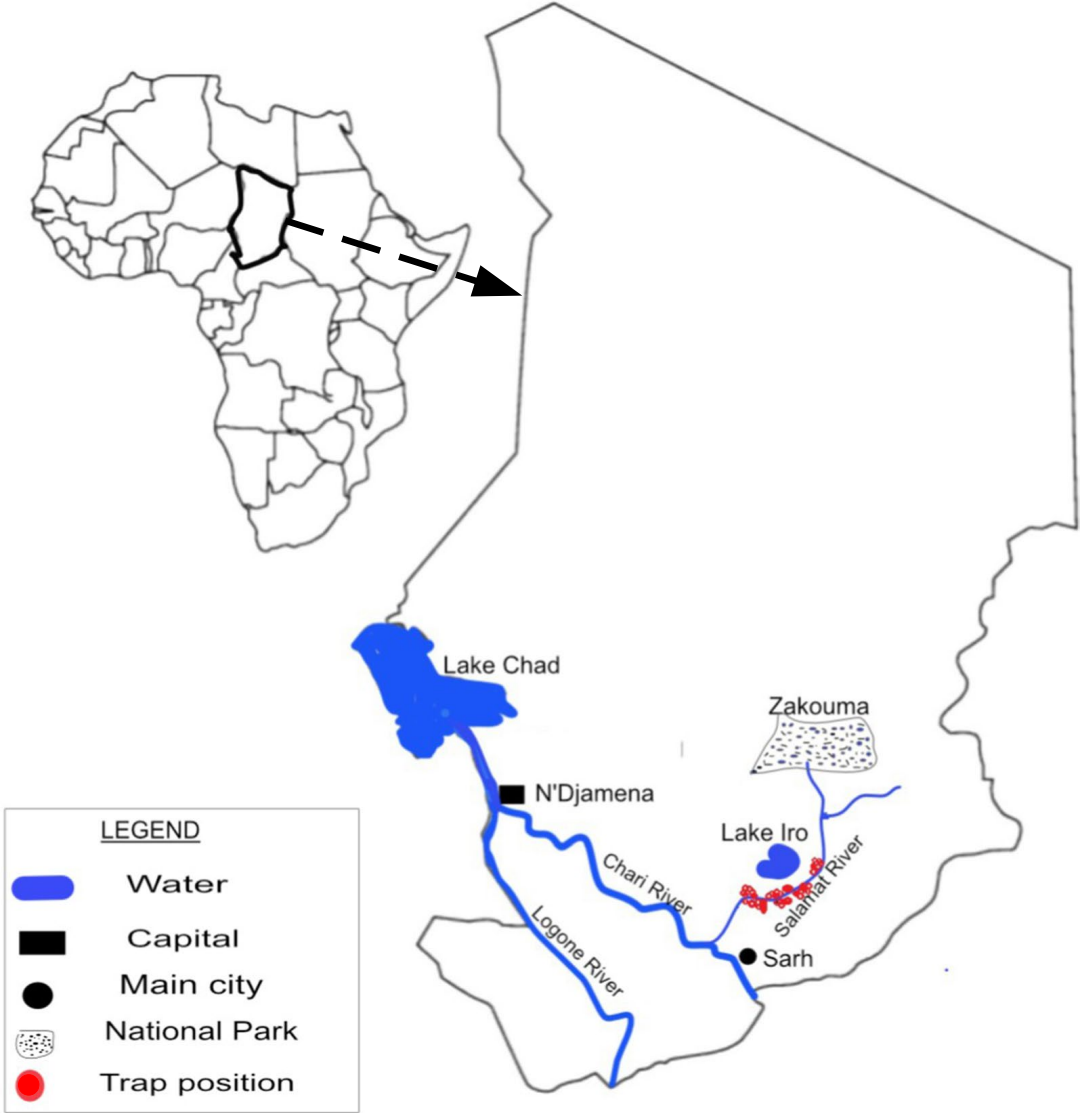
Localization of the area of lake Iro showing the trapping sites

The Lake Iro area (09°59′29.1″N, 019°26′55.7″E) is located 110 km north of Sarh, the capital city of the Moyen Chari Region in southern Chad. It is located in the catchment area (195,000 km^2^) of Bahr Salamat, a seasonally intermittent river, that belongs to the Chari-Logone sub-basin originating from the Darfur Region of Sudan. Lake Iro is close to Zakouma National Park (Fig. 1) . During the dry season, wild animals (antelopes, wild boards, monkeys, warthogs, buffaloes) from Zakouma National Park move in and around the lake Iro area to search for water and grazing area. The hydrographic network of the area is dominated by Lake Iro (surface area about 105 km^2^) and the Bahr Salamate River with its tributaries. The area is characterized by dense shrub forests and floodplains. The maximum annual rainfall is between 800 and 1200 mm [14], the mean annual temperature is 27 °C and the mean annual relative humidity is 50% [15]. The population is estimated at 174,195 inhabitants [16] who practice traditional livestock farming based on pastoralism and agriculture dominated by cereal production.

### Entomological survey

Three entomological surveys were carried out in November 2018, February 2019 and February 2020. During the first survey, 14 biconical traps [17] were set up for 2 days in biotopes favourable to the tsetse fly along the Salamat River and its tributaries. During the second two surveys, 20 traps were set up for 4 consecutive days. The distance between two traps ranged from 100 to 200 m depending on the density of vegetation. For each trap, geographical coordinates were collected using the global positioning system (model GPSMAP® 60CSx; Garmin Ltd., Schaffhausen, Switzerland). At each trapping site, temperature and relative humidity were recorded using a thermohygrometer (EasyLog TH; Lascar, Whiteparish, UK). Flies were collected every 24 h at 9 p.m. During this process, cages containing tsetse flies were collected and transported to the main camp where flies of each trap were counted and morphologically identified to determine the sex and species.

### Collection of tsetse fly organs

From each tsetse fly, the two wings were removed and placed separately in a 1.5-ml dried cryotube for morphometric analyses. The legs, the proboscis and the remaining body (thorax and abdomen) of each tsetse fly were separately put in different 1.5-ml microtubes each containing 200 µl nucleic acid preservative solution (25 mM sodium citrate, 10 mM EDTA and 70% ammonium sulphate). The legs were used for study of the genetic structure of tsetse populations. For each trap that caught < 10 tsetse flies, tsetse organs were collected from all live flies. However, for traps that caught ≥ 10 tsetse flies, the collected flies were subdivided into two groups; tsetse organs were collected in all live flies of one group and the process repeated until no live tsetse fly could be found in the last group. To avoid cross-contamination of organs from two different tsetse flies, dissecting tweezers were decontaminated by incubation in 5% sodium hypochlorite solution for 20 min, followed by a washing step in double-distilled water. The collected organs were stored at 4 °C in the field and conserved at conserved at − 80 °C in the laboratory.

### DNA extraction from the tsetse body

From each tsetse body, DNA was extracted using 5% Chelex-resin (Chelex 100; Bio-Rad, Hercules, CA, USA). Briefly, each tsetse body was removed from the nucleic acid preservative solution and put into a new 1.5-ml microtube. The tsetse body was then crushed with the tip of a capped Pasteur pipette and 100 µl of homogenized Chelex 5% solution was added. After incubation of the microtubes in the thermomixer for 30 min at 56 °C, each microtube was briefly vortexed before another incubation at 95 °C for 5 min. Each microtube was then centrifuged at 10,000 rpm for 1 min. The supernatant or DNA extract was transferred into another microtube and its concentration determined using the NanoDrop 1000 UV-Vis spectrophotometer (Thermo Fisher Scientific, Waltham, MA, USA). DNA extracts were stored at − 20 °C for further analyses.

### DNA extraction from proboscis

Each proboscis was removed from the microtube containing the nucleic acid preserving solution and put into a new 1.5-ml microtube containing 55 µl of DNA extraction solution (mixture of 9.9 µl of proteinase K at 20 mg/m; with 45.1 µL of phosphate buffered saline). Each microtube was centrifuged at 13,400 rpm for 1 min before its first incubation at 54 °C for 1 min, followed by a second incubation at 95 °C for 5 min. Thereafter, each microtube was vortexed and centrifuged at 10,000 rpm for 1 min. DNA extracts were collected and their concentration determined using the NanoDrop 1000 UV-Vis spectrophotometer (Thermo Fisher Scientific). DNA extracts were stored at − 20 °C for further analyses.

### Molecular identification of tsetse flies

The morphological identification of each caught tsetse fly was confirmed by amplifying and sequencing the mitochondrial DNA fragment of the cytochrome* c* oxidase 1 (*COI*) gene as previously described by Dyer et al. [18]. This amplification was done using CO1-sense (5′-TTG ATT TTT TGG TCA TCC AGA AGT-3′) and CO1-non-sense (5′-TGA AGC TTA AAT TCA TTG CAC TAA TC-3′) primers designed by Dyer et al. [18] and performed in a final volume of 25 µl containing 2.5 U of DreamTaq polymerase, 2.5 µl of DreamTaq buffer (10×), 0.2 mM of dNTPs (all provided by Thermo Fisher Scientific), 2 μM of each primer and 1 μl of DNA extract from the proboscis. The amplification programme consisted of an initial denaturation step of 95 °C, 5 min; then denaturation at 94 °C/1 min, annealing at 55 °C/1 min and elongation at 72 °C/1 min for 35 cycles; and a final elongation at 72 °C for 5 min.

At the end of each PCR reaction, 20 μl of PCR products was checked by electrophoresis in a 1.5% agarose gel containing 3 μl G-stain (Serva, Heidelberg, Germany). After completion of the electrophoresis, the agarose gel was stained and the produced visualized under UV light and then photographed. The expected size of the COI amplicons was 930 bp.

Each sample for which a 930-bp DNA fragment was revealed by electrophoresis was selected and the remaining amplicons purified using the GeneJet DNA Purification Kit (Thermo Fisher Scientific) following the manufacturer’s instructions. Each purified* COI* DNA fragment was sequenced by a commercial company (SeqLab, Göttingen, Germany). The sequences obtained were subjected to BLAST search in the National Center for Biotechnology Information (NCBI) database (Genbank) to identify tsetse species.

### Molecular identification of trypanosome species

Different trypanosome species and subspecies were identified from the proboscis and body of each tsetse fly by amplifying the internal transcribed spacer 1 (ITS1) fragment of the rDNA of trypanosomes as described by Adams et al. [19]. After this amplification, specific identification of each trypanosome species or subspecies was performed by sequencing the amplified ITS1 DNA fragment and comparing the obtained sequences with those available in the gene bank.

### Amplifications of ITS1 DNA fragment of trypanosomes

The amplification of the ITS1 DNA fragment was performed using a nested PCR as described by Adams et al. [19]. In the first PCR, the outer generic primers ITS1-Out-sense (5′-TGC AAT TAT TGG TCG CGC-3′) and ITS1-Out-non-sense (5′-CTT TGC TGC GTT CTT-3′) were used, and the amplification reactions were performed in a final volume of 25 µl containing 2.5 U of DreamTaq polymerase (5U/µl), 2,5 µl DreamTaq green buffer (10×), 0.2 mM of dNTPs (Thermo Scientific Scientific), 2 µM of each outer primer and 1 µl of DNA extract from the tsetse fly body or 5 µl of DNA extract from the proboscis. The amplification programme for the first PCR consisted of one cycle of initial denaturation at 95 °C for 5min﻿; then denaturation at 94 °C/1 min, annealing at 54 °C/ 30 s and elongation at 72 °C/30 s for 30 cycles; and a final elongation at 72 °C for 5 min.

PCR products of the first PCR were diluted (1:1000 dilution), and 1 µl was used as the DNA template for the nested PCR. For this PCR, 2 µM of each inner primer (ITS1-In-sense: 5′-TAG AGG AAG CAA AAG-3′; ITS1-In-non-sense: 5′-AAG CCA AGT CAT CCA TCG-3′) was used; the amplification reactions were carried out using the same conditions described above for the first PCR.

Amplified products of the second PCR were checked by electrophoresis in 1.5% agarose gel as described above. Trypanosome species were identified on the basis of length polymorphism of the ITS1 fragments. *Trypanosoma congolense* strains (*T. congolense* forest and savannah) are expected to generate DNA fragments of about 650 bp while the expected sizes for *T. brucei* (*s.l.*) and *T. vivax* are around 400 and 150 bp, respectively.

### Sequencing of amplified DNA fragments of the ITS1 of different trypanosomes

To confirm the results generated on the basis of length polymorphism of the ITS1 fragment, five representative amplicons for each size were purified using the GenJet Purification Kit (Thermo Fisher Scientific) following the instructions of the manufacturer. All amplicons with a size of ≥ 400 bp were sequenced directly by a commercial company (SeqLab) while those < 400 bp were cloned before sequencing.

### Cloning and sequencing of amplified ITS1 fragments

Purified PCR products were cloned into the linearized pJET 1.2/blunt vector using the CloneJET PCR procedure (Thermo Fisher Scientific) according to the manufacturer’s instructions. Recombinant colonies were identified by PCR in which a small portion of the colony was scraped with a sterile pipette tip and each scraped colony used as DNA template for a PCR reaction in which the ITS1 fragment was amplified using the primers described above. Each recombinant colony was picked up and cultured overnight at 37 °C in LB medium supplemented with ampicillin (100 μg/ml). The bacterial culture was centrifuged at 4500 *g* for 15 min at 4 °C, following which the pellet was collected and the plasmid DNA purified using the GeneJET Plasmid MiniPrep Kit (Thermo Fischer Scientific) according to instructions of the manufacturer. Plasmid DNA containing DNA fragments of ITS1 was sent to a commercial company (SeqLab) for sequencing.

Geneious Pro version 5. 5. 9 software was used to store, organize and analyse the obtained sequences. Trypanosome species or subspecies was identified by subjecting each ITS1 sequence to BLAST search in the NCBI database (Genbank).

### Data analysis

Statistical analyses were performed using R software [20]. The chi-squared (*χ*^2^) test was used to compare the infection rates of different trypanosomes. Comparisons were made between infections in the tsetse body and those in the proboscis as well as between infections according to the sex of tsetse flies. Differences were considered to be significant at* P*-values < 0.05.

## Results

A total of 617 tsetse flies were trapped during the three surveys: 12 in November 2018, 323 in February 2019 and 282 February 2020 (Table 1). The overall apparent density of tsetse flies per trap per day (ADT) was 2.9, with ADT = 0.4 in November 2018, 4.0 in February 2019 and 3.5 in February 2020 (Table 1). From these tsetse flies, 32 (5.2%) were tenerals (tsetse flies that have not yet taken a blood meal and consequently have not been exposed to trypanosome infections). We selected 359 non-teneral flies and removed their proboscis, legs and wings as described in section Collection of tsetse fly organs. The remaining body and the proboscis of each tsetse fly were subjected to molecular identification of different trypanosomes. Sex distribution showed that 68.6% of all flies captured were males while 31.4% were females, giving a sex ratio of 2.2.

**Table 1:** Entomological data according to sampling periods

Sampling period	Number of captured tsetse	Number of teneral flies (%)	Number of tsetse selected (%)	Number of traps	ADT	Number of males (%)	Number of females (%)	Sex ratio
December 2018	12	0 (0)	12 (100)	14	0.4	7 (58.3)	5 (41.7)	1.4
February 2019	323	18 (5.6)	222 (68.7)	20	4.0	231 (71.5)	92 (28.5)	2.5
February 2020	282	14 (4.9)	125 (44.3)	20	3.5	185 (65.6)	97 (34.4)	1.9
Total	617	32 (5.2)	359 (58.2)	54	2.6	423 (68.6)	194 (31.4)	2.2

*ADT* Apparent density of tsetse flies per trap per day

### Diversity of tsetse flies

Molecular identification of tsetse flies was carried out on the 359 tsetse flies from which the proboscis, legs, wings and tsetse bodies were collected. From the proboscis of each of these 359 tsetse flies, a COI DNA fragment of around 930 bp was successfully amplified and sequenced. The comparison of sequences generated from different tsetse flies revealed three tsetse species in the area of Lake Iro. *Glossina m. submorsitans* was the dominant tsetse species and constituted 96.1% of the 359 tsetse flies that were sequenced; *Glossina f. fuscipes* represented 3.06% and *G. tachinoides* represented only 0.8% (Table 2). Of the 345 tetse flies identified as *G. m. submorsitans*, 240 were male (69.5%) and 105 were female (30.4%). For the 11 *G. f. fuscipes* identified, seven (63.6%) were male and four (36.3%) were female. All three *G. tachinoides* identified were female.

**Table 2:** Type and number (prevalence) of trypanosome infections according to tsetse species

Tsetse species (NTA)	Type of infections	Body parts	Trypanosome species	NIT (%)
*Glossina morsitans submorsitans* (345)	Single infections	Proboscis or tsetse body	*T. simiae* (64 b and 4 p)	68 (19.7)
			TCS (39 b and 16 p)	55 (15.9)
			*T. vivax* (7 b and 32 p)	39 (11.3)
			*T. goffreyi* (30 b and 4 p)	34 (9.8)
		Total of single infections		196 (56.7)
	Mixed infections	Tsetse body	*T. simiae* and TCS	2(0.6)
			*T. simiae* and *T. godfreyi*	1 (0.3)
			*T. godfreyi* and TCS	2 (0.6)
			*T. simiae* and *T. vivax*	1 (0.3)
			Mixed infections in tsetse body	6 (1.7)
		Proboscis and tsetse body	*T. simiae* (b) and *T. vivax* (p)	5 (1.4)
			TCS (b) and *T. vivax* (p)	3 (1.4)
			*T. godfreyi* (b) and *T. vivax* (p)	2 (0.6)
			Mixed infections in proboscis and tsetse body	10 (2.9)
		Total of mixed infections		16 (4.6)
	Total of infected *G. m. submorsitans*			212 (61.4)
*G. fuscipes fuscipes* (11)	Single infections (proboscis (p) or tsetse body (b)		*T. simiae* (b)	2 (18.2)
			TCS (b)	3 (27.2)
			*T. vivax* (1 b and 1 p)	2 (18.2)
			*T. goffreyi* (b)	1 (9.1)
	Total of infected *G. f. fuscipes*			8 (72.7)
*G. tachinoides* (3)	Single infections (Proboscis or tsetse body)		TCS (b)	1 (33.3)
			*T. vivax* (p)	1 (33.3)
	Total of infected *G. tachinoides*			2 (66.6)
Total (359)				222 (61.8)

*NIT* Number of infected tsetse flies, *NTA *Number of tsetse flies analysed, *TCS* *Trypanosoma congolense* savannah, *p* proboscis, *b* tsetse body

### Diversity of trypanosome species

**Fig. 2:**
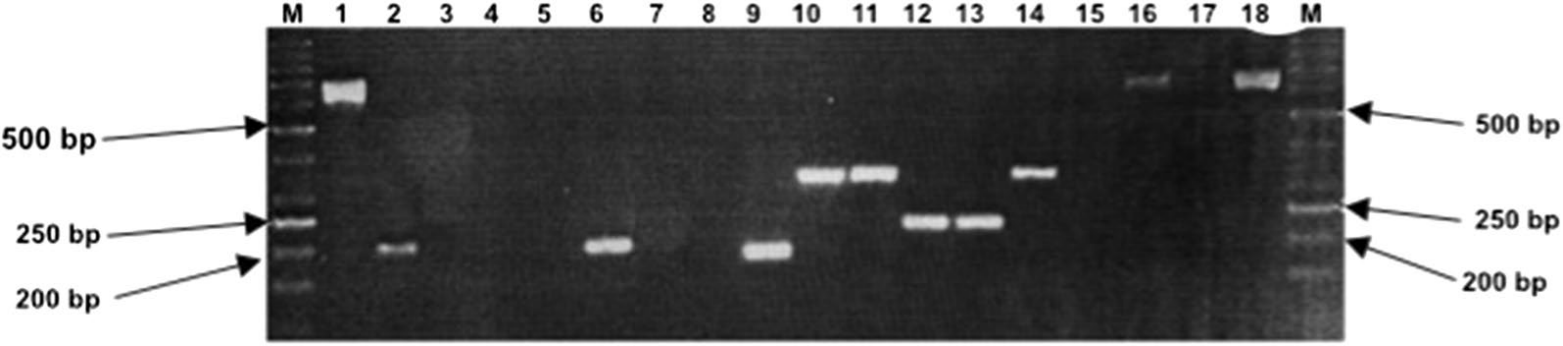
Example of agarose gel illustrating the electrophoretic profiles generated from the amplification of the internal transcribed spacer 1 fragment of different trypanosome species. Lanes:* M* Marker GeneRuler 50 bp Ladder (Thermo Fisher Scientific),* 1*,* 16* *Trypanosoma congolense*,* 2*,* 6*,* 9* *T. vivax*,* 10*,* 11*,* 14* *T. simiae*,* 12*,* 13* *T. godfrey*,* 17* negative control without DNA template,* 18* positive control (genomic *T. congolense* DNA from infected cattle)

Results of length polymorphism analysis (Fig. 2) and sequencing of ITS1 fragments revealed the presence *T. congolense* savanah, *T. vivax*,* T. simiae* and *T. godfreyi* in tsetse flies caught in the area of Lake Iro. From the randomly selected 359 tsetse flies that were subjected to molecular identification of trypanosomes in the proboscis and the tsetse body, 222 (61.8%) were found with at least one trypanosome infection. Of the 345 *G. m. submorsitans* analyzed*,* 212 (61.4%) were infected, of which 196 (56.7%) and 16 (4.7%) were found with single and mixed infections of trypanosomes, respectively (Table 2). For the 196 (56.7%) *G. m. submorsitans* that carried trypanosomes species in their body or their proboscis, the parasites identified included *T. simiae* (19.7%), followed by *T. congolense* savannah (15.9%), *T. vivax* (11.3%) and *T. godfreyi* (9.8%).

Eight (72.7%) *G. f. fuscipes* and two (66.6%) *G. tachinoides* were found with trypanosome infections in the proboscis and/or the tsetse body. In *G. f. fuscipes*, *T. congolense* savannah (27.3%) was the most prevalent trypanosome species, followed by *T. simiae* (18.2%), *T. vivax* (18.2%) and *T godfreyi* (9.1%). In *G. tachinoides, T. congolense* savanah and *T. vivax* were detected in the proboscis and the tsetse body with an identical infection rate of 33.3% (Table 2).

Male and female *G. m submorsitans* were found with all trypanosome species identified in this study. Comparison of the trypanosome infections revealed that there was no significant difference between males and females for infection with *T. congolense* savanah (*χ*^2^ = 0.7665; *P* = *0.38*),* T. vivax* (*χ*^2^ = 0.1035;* P* = 0.75),* T. godfreyi* (*χ*^2^ = 0.1035;* P  *= 0.75),* T. simiae* (*χ*^2^ = 1.6028;* P  *= 0.21) and all trypanosome species taken together (*χ*^2^ = 0.0067; *P* = 0.9). Given the small number of *G. f. fuscipes* (11 individuals) and *G. tachinoides* (3 individuals), no comparison of trypanosome infection rates between sex and tsetse species was performed.

### Trypanosome infections in the proboscis and tsetse body

All trypanosome species identified in this study were found in both the proboscis and the body of *G. m. submorsitans.* Trypanosome infections were detected in 56 (16.2%) proboscises and 140 (40.6%) bodies of 345 *G. m. submorsitans.* In proboscises, *T. vivax* was the most prevalent trypanosome species (9.3%), followed by *T. congolense* savanah (4.6%) and then *T. simiae* and *T. godfreyi* with an infection rate of 1.2% each. In the tsetse body, *T. simiae* was the most prevalent trypanosome species (18.5%), followed by *T. congolense* savanah (11.3%), *T. gofreyi* (8.7%) and *T. vivax* (2.0%). Comparison of the trypanosome infection rates in *G. m. submorsitans* revealed a significant difference (*χ*^2^ = 50.283; *P* < 0.00001) between infections in the probosis and those of the body. The infection rate of *T. vivax* was significantly higher (*χ*^2^ = 16.986; *P* < 0.00001) in the proboscis than in the body of *G. m. submorsitans*.

In *G. f. fuscipes,* all trypanosome species were detected in tsetse bodies while only *T. vivax* was identifed in the proboscis. Of the 11 *G. f. fuscipes* studied, only one (9.1%) infection of *T. vivax* was found in the proboscis while seven (63.6%) tsetse bodies were infected by the four species of trypanosomes. *Trypanosoma congolense* savanah (27.3%) was the most prevalent trypanosome species, followed by *T. simiae* (18.2%), then *T. vivax* and *T. godfreyi* (both an infection rate of 9.1%). For *G. tachinoides*, only *T. congolense* savanah was detected in the tsetse body and *T. vivax* in proboscis.

### Mixed infections of different trypanosomes

No mixed infection was detected in the proboscis of all tsetse species analysed nor in the body of *G. f. fuscipes* and *G. tachinoides.* Only double infections were detected, and they included six (1.7%) mixed infections, appearing in the tsetse body only, and ten (2.9%) others in both the proboscis and tsetse body. The six mixed infections that were detected only in the tsetse body included two (0.6%) tsetse flies with *T. simiae* and *T. congolense* savanah, two (0.6%) with *T. simiae* and *T. godfreyi*, one (0.3) with *T. godfreyi* and *T. congolense* savanah, and one (0.3%) with *T. simiae* and *T. vivax*. For the ten tsetse flies with trypanosome infections in both the proboscis and tsetse body, five (1.4%) had *T. simiae* in the tsetse body and *T. vivax* in the proboscis; three (0.9%) had *T. congolense* savannah in the tsetse body and *T. vivax* in the proboscis and two (0.6%) had *T. godfreyi* and *T. vivax* respectively in the tsetse body and the proboscis (Table 2).

## Discussion

Although several investigations have been undertaken in sleeping sickness foci of Chad to generate data on tsetse fauna as well as trypanosome infections in human and animals [12, 13, 21, 22], the situation of tsetse-transmitted trypanosomiases remains unknown in several tsetse-infested areas. This study was designed to fill this gap by identifying tsetse species and trypanosomes infecting these flies for the overarching goal of improving epidemiological knowledge on AAT in the southern area of Chad. Despite the limitation that less than 60% of the tsetse flies caught were identified, the capture of *G. m. submorsitans*, *G. f. fuscipes* and *G. tachinoides* in the south of Chad confirms the results of recent studies demonstrating the presence of these tsetse flies in this area [12, 13] as well as those of much older studies carried out more than 50 years ago [24, 25].

The low apparent density of tsetse flies recorded in November 2018 compared to February 2019 and 2020 (dry season) could be explained by some bioclimatic conditions that vary between the trapping periods. Indeed, the month of November corresponds to the end of the rainy season, and buried pupae likely did not survive submersion resulting from the increased level of flowing water and overflow of rivers in the Chari-logone sub-basin. Moreover, the number of rainy days in November is an important determinant factor of tsetse fly density as the movements of tsetse flies are limited in time and space during this period month, especially during the rainy days, thus restricting access to resting and/or emerging sites. Also, since rainy days are not favourable in terms of tsetse flies moving towards the traps, trapping during rainy days, as was conducted during the present study, is an additional factor explaining the low density of tsetse flies caught at the end of the rainy season in November.

The large number of male *G. m. submoritans* (68.6%) compared to females (31.4%) contradict previous results of a study in Nigeria where approximately equal numbers of males and females were reported for the same tsetse species [25]. One explanation for the low number of females recorded in the present study is that in harsh conditions, most flies are hidden in vegetation and restrict their movements. In such conditions, tsetse fly movements are probably extremely reduced and, interestingly, females have been reported to be active only for 5 min per day [26]. Moreover, the sampling device could introduce some bias in the number of males or females caught because the sex ratio of caught tsetse flies can greatly vary according to the type of trap [17].

The identification of *G. m. submorsitans* as the main cyclic vector of African trypanosomes in the area of Lake Iro is in agreement with results obtained in the Yankari Game Reserve and Lake Kainji National Park of Nigeria [25]. Known as the savannah tsetse species, the predominance of *G. m. submorsitans* in our study site could be explained by the bioclimatic conditions, with the wooded savannah of the area offering a favourable environment for its life-cycle. Moreover, *G. m. submorsitans* is largely dependent on big game for blood meals [27], and the proximity of Lake Iro to the Zakouma National Park [28, 29], which contains large populations of wild animals that move towards Lake Iro during the dry season, provide abundant animals for the blood meals. This tsetse species appears to be the most important vector of AAT in the locality of Lake Iro.

The lower number of *G. f. fuscipes* and *G. tachinoides* caught could be explained by the unfavourable bioclimatic conditions for their development. Known as riparian species due to their high preference for areas with a high soil moisture content, such as mangroves, lake shores and gallery forests along rivers, the bioclimatic conditions (type of soil, temperature, humidity) around the Lake Iro does not offer favourable environments for development of these tsetse species. Despite their low number, it is important to note that *G. f. fuscipes* and *G. tachinoides* may play a role in the epidemiology of AAT, as evidenced by the identification of several pathogenic trypanosome species in these tsetse fly species. These species could, therefore, facilitate the transmission of pathogenic trypanosome species to vertebrate hosts.

Four trypanosome species, namely *T. simiae*, *T. congolense* savannah, *T. vivax* and *T. godfreyi*, were identified in all three tsetse fly species caught in this study, indicating that each of these flies could play a role in the epidemiology of AAT in the area of Lake Iro. Although the numbers of *G. f. fuscipes* and *G. tachinoides* caught were low, the trypanosome infection rate in each tsetse species was high (56.9% for *G. m. submorsitans*, 72.7% for *G. f. fuscipes* and 66.6% for *G. tachinoides*)*.* Trypanosome infection rates obtained in the present study are higher than those reported by in earlier studies [9, 30]**.** Although we did not perform any analysis of blood meals in this study, it is likely that *G. m. submorsitans* in the area of lake Iro tend to feed mainly on wild animals since, as mentioned above, the Zakouma National Park is located relatively close to this lake. In this context, the transmission cycle of trypanosomes in this area is predominantly “wild animal/tsetse/wild animal”. The high infection rates reported in tsetse flies indicate a high transmission of African trypanosomes between tsetse flies and wild animals; however, cattle also move in and around Lake Iro, and thus are exposed to tsetse bites and consequently to trypanosome infections. These cattle are therefore at high risk for trypanosome infections. Moreover, the detection of *T. congolense* savannah in the three tsetse species caught in this study testifies not only its high circulation between tsetse and mammals, but also to its potential impact on infected cattle. As such, the results of this study suggest that the numerous wild and domestic animals residing in or moving into/out of the area of Lake Iro could be potentially infected by different trypanosome species. AAT could therefore be considered as a serious risk for animal health and animal breeding in the area of Lake Iro.

Comparison of the proboscis and tsetse body in terms of trypanosome infection revealed that there were a higher number of infections in the tsetse body than in the proboscis, possibly due to the presence of trypanosome infections in different organs, such as the migdut and/or the salivary glands. These results are in agreement with those reported in Nigeria where the proboscises of different tsetse species were less infected than salivary glands and guts [30, 31]. Nevertheless, the possibility of having remaining blood meals with trypanosome infections in tsetse midguts cannot be ruled out. As no dissection of tsetse flies was performed to determine which tsetse organ was infected by each trypanosome species, it is not possible to speculate on immature or mature infections of different trypanosomes. The trypanosome infections reported in tsetse bodies could be assimilated into immature and/or mature infections depending on the trypanosome species involved. For example, the identification of *T. vivax* in the proboscis testifies to a mature infection because *T. vivax* migrates directly from the midgut* via* the proventriculus to the proboscis where it develops into the trypomastigote and metacyclic forms [32]. The high *T. vivax* infection rate observed in the proboscis (9.3%) compared to the tsetse body (2.0%) could be explained by the fact that its developmental cycle occurs predominantly in the proboscis. The detection of *T. vivax* in the proboscis indicates a high probability of its transmission to mammals during blood meals, but its detection in the tsetse body is strange because its developmental cycle is normally restricted to the mouthparts. Nevertheless, the detection of *T. vivax* in the tsetse body can be explained in part by the residual blood meals taken on mammals infected by *T. vivax*, or by recent infections in which these parasites are in the process of migrating towards the proboscis to complete their developmental cycle. In this study, the presence of DNA from *T. vivax* or other trypanosome species was taken as evidence of an active infection of this trypanosome species. However, it is important to mention that DNA from dead trypanosomes can be amplified and detected in the absence of any active trypanosome infection.

Compared to the infection rate in the proboscis, the high infection rates reported in the tsetse body for *T. simiae* and *T. godfreyi* are in line with their developmental cycles. These results indicate not only a probable high transmission of these trypanosomes, but also the presence of suitable vertebrate hosts for these parasites. Although pigs have been reported to be suitable hosts for *T. simiae*, their absence in the locality of Lake Iro suggests the presence of other vertebrate hosts, such as wild boar and warthogs that have been frequently observed during entomological surveys. *Trypanosoma simiae* is recognized to be highly pathogenic for pigs [32, 34], but the active transmission of this species is of less epidemiological value for AAT in the study area because inhabitants and visiting farmers of Lake Iro are mostly Muslim. In this context, pig farming is not developed and, consequently, infections due to *T. simiae* are probably of limited epidemiological importance for animals breeding in this locality. While the importance of *T. godfreyi* as a pathogen of AAT remains unclear, the results of this study indicate an active transmission of this parasite in the area of Lake Iro.

The detection of *T. congolense* savanah both in the tsetse body and the proboscis indicates immature and mature infections of this trypanosome. The high infection rate of *T. congolense* savanah in tsetse bodies (11.3%) compared to proboscises (4.2%) is in agreement with results reported in other tsetse species [9, 31]. Taken together, these results indicate the predominance of immature infections of *T. congolense* in tsetse flies in the locality of Lake Iro. These immature infections could be explained by the need for *T. congolense* savannah, following a blood meal on infected mammals, to first establish in the midgut as the procyclic form before continuing its development and migration to the proboscis in the mature form ready to be transmitted to uninfected mammals. However, the bottleneck that has been observed in the life-cycle of trypanosomes from their establishment in the midgut to their maturation in the salivary glands or the proboscis depends on the trypanosome species, possibly also explaining the low infection rate of trypanosomes in the proboscis compared to the tsetse body. Taken into consideration the virulence of *T. congolense* savanah and *T. vivax* and their real impact on animal health [35, 36], the detection of these trypanosomes in *G. f. fuscipes, G. tachinoides* and *G. m. submorsitans* shows that cattle in the locality of Lake Iro and those passing through there during transhumance are potentially at higher risk for AAT. The high infection rates reported in the proboscis indicate mature infections of *T. vivax* and *T. congolense* savanah, demonstrating that each tsetse fly carrying such infections will easily transmit trypanosomes to healthy mammals during each blood meal. Investigations on tsetse distribution and trypanosome infections in livestock living in and around the area of lake Iro are becoming important for the understanding of the current epidemiological situation of AAT, and also for the designing and implementing disease control operations. While waiting for political and financial commitments at the national level, and also for additional entomological data as well as data on trypanosome infections in livestock, some control measures can be initiated in some risky biotopes by setting up traps and/or screens or by spraying insecticides to kill tsetse flies to prevent trypanosome infections, especially in livestock that pass through this area. Furthermore, regular treatment of infected livestock with trypanocides could be planned to reduce the number of infected animals and, consequently, the probability of tsetse flies feeding on such animals and faciliate trypanosome transmission.

Our results showing no significant difference in the trypanosome infection rates according to sex are in agreement with experimental infections [38, 39]. However, in natural tsetse populations, several studies have reported female flies to be more infected than their male counterparts because females live longer [31, 40]. As already mentioned above, the high temperature recorded in the area of Lake Iro probably induced a high mortality rate among females, thus explaining the similar trypanosome infection rates observed in male and female flies.

Although no information on mixed infections involving mature infections of different trypanosome species can be inferred, the detection of both *T. vivax* and *T. congolense* savanah in the proboscis of one tsetse fly indicates the possibility of these two highly pathogenic trypanosomes being transmitted to one animal during a single blood meal. If such a transmission occurs, could each of these trypanosome species have the potential to develop in the vertebrate host? Some mixed infections detected in tsetse flies could result from a single blood meal on mammals infected by different trypanosome species. It has been demonstrated that among infected tsetse flies, a mature infection will develop in only a proportion of those harbouring immature trypanosome infections [31, 41]. All trypanosome species detected in tsetse flies harboring mixed infections were unable to develop to the mature form and, consequently, could not be transmitted to mammals. The high number of tsetse flies with double infections, namely *T. congolense* savannah and *T. simiae*, or *T. congolense* and *T. godfreyi*, suggests not only high circulation of these trypanosome species but also their predominance in mammals.

### Conclusion

The results of this study reveal the presence of *G. m. submorsitans*, *G. f. fuscipes* and *G. tachinoides* in the area of Lake Iro in the south of Chad. *Glossina m. submorsitans* appeared to be the main vector of African trypanosomes in this area. Several pathogenic trypanosomes, including *T. vivax*,* T. simiae*,* T. godfreyi* and *T. congolense* savanah can be transmitted by different tsetse species. These findings highlight the risk of AAT for livestock breeding and the importance of assessing trypanosome infections in livestock in the area of Lake Iro.

## Data Availability

All data generated and/or analyzed during this study are included in this article.

## Abbreviations

AAT: Animal African trypanosomiasis
ADT: Apparent density of tsetse flies per trapping
*COI*: Cytochrome* c* oxidase 1 gene
HAT: Human African trypanosomiasis
ITS1: Internal transcribed spacer 1
